# Temporal trends in behavioral risk and protective factors and their association with mortality rates: results from Brazil and Argentina

**DOI:** 10.1186/s12889-020-09512-9

**Published:** 2020-09-11

**Authors:** Leonardo Pozza Santos, Fernanda de Oliveira Meller, Valeria Romina Amann, Antônio Augusto Schäfer

**Affiliations:** 1 Nutrition College, Federal University of Pampa, Luiz Joaquim de Sá Britto, s/n,, Itaqui, 97650000 Brazil; 2 Postgraduate Program in Public Health, University of Southern Santa Catarina, Criciúma, Brazil; 3 Scientific and Training Subcommittee of the Nutritionists College, Province of Misiones, Argentina

**Keywords:** Nutrition surveys, Population groups, Latin America

## Abstract

**Background:**

Despite available information on trends in behavioral factors for Brazil and Argentina, little is known about the association of these trends with mortality. Understanding this association is important to avoid early deaths. Therefore, we aimed to evaluate temporal trends in behavioral risk and protective factors in Brazil and Argentina, and to assess their association with overall and cause-specific mortality rates.

**Methods:**

Ecological study with data from two population surveys from Brazil and Argentina. Weighted prevalence of tobacco smoking, excessive alcohol consumption, fruit and vegetable consumption and physical activity for the 27 Brazilian state capitals and for the 23 Argentinean provinces were used as behavioral factors. Information on overall mortality as well as cardiovascular diseases and cancer mortality for the year 2015 was collected from national mortality banks of both countries. Estimated prevalence rates were used to describe trends in behavioral factors from 2006 to 2014 in Brazil, and from 2005 to 2013 in Argentina, while Pearson’s correlation and linear regression models were used to assess their association with overall and cause-specific mortality rates.

**Results:**

Brazil presented improvements in behavioral risk and protective factors: sharp decrease in tobacco smoking prevalence (from 15 to 9%), increase in regular fruit and vegetable consumption (from 28 to 36%), and increase in physical activity (45 to 51%). In Argentina, results were more disappointing: small reduction in tobacco smoking (from 55 to 50%) and decrease in physical activity (from 55 to 45%). In both countries, excessive alcohol consumption remained stable, with increase only among women. The association between behavioral factors and mortality showed that in those Brazilian capitals with higher prevalence of regular consumption of fruits and vegetables, there were lower overall mortality rates. Stratification by gender revealed that significant results were only found among women.

**Conclusion:**

Prevalence of regular consumption of fruits and vegetables increased in Brazilian capitals and was associated with lower overall mortality rate, suggesting a positive impact of Brazilian policies to improve dietary intake patterns on its population’s mortality. Approaches focusing on behavioral factors are especially needed in Argentina to reach similar results of those seen in Brazil.

## Background

Noncommunicable chronic diseases (NCDs) account for 71% of global deaths and more than three-quarters of all deaths in low- and middle-income countries (LMIC) [1, 2]. Most NCDs deaths are caused by cardiovascular diseases (CVD) (17.9 million people annually) and cancers (9.0 million) [1]; both of them are health issues caused by genetic, physiological, environmental and behavioral factors [1, 3]. NCDs also account for the majority of deaths (most notably CVD and cancers) in Latin America and the Caribbean [4], a region that comprises fifty independent countries with 640 million inhabitants [5].

In Argentina and Brazil, two of the biggest Latin American countries which, along with Colombia and Mexico, have two-thirds of the Latin American population and three-quarters of the regional gross domestic product (GDP) [5], NCDs are also responsible for a large proportion of total deaths. In Argentina, NCDs were the reason for 79.3% of all deaths in 2009 [6], and their main causes were CVD, cancers, diabetes and respiratory illnesses [7–9]. Brazil faces a similar scenario; NCDs have been leading causes of years lived with disability and deaths in the last three decades, and in 2016 the two main causes of years of life lost and deaths were CVD and cancers [10].

NCDs are mostly associated with behavioral factors, such as tobacco smoking, excessive alcohol consumption, unhealthy feeding habits and physical inactivity. Despite global efforts made to prevent and control these risk factors [11–15], their occurrences remain high, especially in LMIC [11, 16–18]. For example, global exposure to smoking declined by more than 25% in the last decades [16], although the number of smokers remains high in some regions as a result of demographic growth. Additionally, approximately 80% of the 1.1 billion smokers worldwide live in LMIC [19]. As regards excessive alcohol consumption, total per capita alcohol consumption has increased in people aged ≥15 years old and is expected to increase in the Americas, Southeast Asia, and the Western Pacific, reaching 6.6 l in 2020 and 7.0 l by 2025 [11].

Even with slight improvements in eating habits and physical activity (PA) in recent years, consumption of unhealthy foods and sedentarism are still high. A recent study showed a global increase in ultraprocessed food and drink product sales, mainly in LMIC [20]. In Brazil, there was an increase in consumption of fruits and vegetables (F&V) from 2008 to 2015, but it started to fall again in 2016 [21]. Concerning PA, 23% of global adult population is insufficiently physically active [12], and this rate is even higher in the Americas, reaching almost 50% of the adult population [17].

Considering that behavioral risk factors for NCDs are modifiable, an important way to prevent and control these diseases is to focus on reducing their prevalence. Moreover, monitoring progress and trends of behavioral risk and protective factors is important to guide policy and priorities in order to avoid early deaths [1]. Therefore, the aim of this study was to evaluate temporal trends in behavioral risk and protective factors in Brazil and Argentina, and to assess their association with overall and cause-specific mortality rates.

## Methods

### Design and sample

This is an ecological study with data from two cross-sectional population surveys that have taken place in Brazil and Argentina. It evaluated 27 Brazilian cities (the 26 state capitals and the Federal District) and 23 Argentinean provinces (the Federal District was included in the Buenos Aires province). Both studies occur regularly in each country and are aimed at investigating risk factors for NCDs.

The Brazilian survey, called Surveillance of Risk and Protective Factors for Chronic Diseases (‘*Vigitel*’ in the Brazilian acronym), is a population-based survey of adults (18 years or older) who live in the 26 Brazilian state capitals and the Federal District and have a landline telephone. To be eligible to participate in the study, the selected adult must live in the corresponding selected house. Since 2006, Vigitel has been conducted annually and has interviewed more than 40,000 individuals every year.

Vigitel uses a sampling process that is probabilistic and stratified into two steps: random selection of house landline telephone numbers and random selection of a person to be interviewed. In this study, we used data from 2006 and 2014 Vigitel editions. In order to ensure a maximum error of 2% points for prevalence estimates of the study risk factors, more than 2000 telephone interviews were conducted in each city in 2006, in a total of 54,369 individuals. The number of interviews dropped slightly in 2014; more than 1500 interviews in each city, in a total of 40,853 individuals [22, 23].

The Argentinean survey, called National Survey of Risk Factors (‘*ENFR*’ in the Argentinean acronym), is held every 4 years in the 23 Argentinean provinces and in the Federal District. It is also a population-based survey of adults, but unlike Vigitel, its interviews are conducted in selected households but not by phone. The sampling process of this survey is conducted in four steps: the first three steps are part of the *Muestra Maestra Urbana de Vivendas da República Argentina*, which is a probabilistic selection of areas with 2000 inhabitants or more. The last step is the random selection of an adult to answer the questionnaire. In our study, we used information from 2005 and 2013 ENFR editions. In both ENFR editions used here, more than 1000 interviews were conducted in each Argentinean province, in a total of more than 40,000 individuals [8, 24].

### Behavioral risk and protective factors

We included tobacco smoking and excessive alcohol consumption as risk factors of our study. The classification of tobacco smoking was based on individuals who self-reported as smokers at the time of the interview. Excessive alcohol consumption was considered as the single-time consumption, at least once in the last 30 days, of five or more doses of alcohol for men and four or more doses for women.

PA and regular consumption of F&V were the behavioral protective factors included in our study. We considered as physically active those individuals who have reported 150 min or more of any PA per week. This classification was based on PA recommendations for this age group [25]. Regular consumption of F&V was defined as the consumption of at least one portion of F&V in five or more days a week. Regular consumption of F&V was only assessed in Brazil because of conceptual differences in the questions designed to evaluate this information between 2005 and 2013 in Argentina, which did not enable a comparison.

For both behavioral risk and protective factors, prevalence in each capital/province was defined as the total number of people classified with a particular condition, divided by the total number of individuals and multiplied by 100. We also calculated gender-stratified prevalence of behavioral risk and protective factors (total number of men/women classified with a particular condition, divided by the total number of men/women and multiplied by 100). All prevalence rates were estimated using weighting factors, considering the complex sampling of both surveys, using the command ‘*svy*’ in Stata.

### Socioeconomic and demographic factors

Information about socioeconomic and demographic characteristics associated with exposures and outcomes were included as potential confounders of our study. As socioeconomic factors, we included the Human Development Index (HDI) and educational level of each capital/province. Data on HDI were collected from the Brazilian Institute of Geography and Statistics for Brazil (for the year 2000) [26], and from the National Report on Human Development for Argentina (for the year 2001) [27]. Educational level was defined with a basis on the estimated prevalence of individuals who completed high school, at least. As demographic factors, we included the average age of respondents as well as the distribution of gender in each capital/province.

### Overall and cause-specific mortality rates

Information on overall and cause-specific mortality in 2015 was collected from national mortality banks of both countries. The inclusion of mortality information for the year 2015 was made considering a small latency period between the time of exposure to behavioral factors (from 2006 to 2014 in Brazil, and from 2005 to 2013 in Argentina) and the outcome, which could increase robustness to assess causality. In Brazil, data were collected from the Mortality Information System of the Ministry of Health. In Argentina, data were gathered from the Department of Statistics and Health Information of the Ministry of Health. In both countries, we collected information on mortality by place of residence and by age group.

Information on cause-specific mortality was determined for deaths from CVD and cancers. We included these two cause-specific mortalities since they are the main causes of deaths in Brazil and Argentina. Mortality for CVD included the following codes of the 10th revision of the International Classification of Diseases (ICD-10) [28]: I10-I15 (hypertensive diseases), I20-I25 (ischemic heart diseases), I49-I50 (heart failure), I60-I69 (cerebrovascular diseases), I70 (atherosclerosis) and I74 (embolism and arterial thrombosis). Mortality for cancers included all the codes referent to neoplasm and tumors (from C00 to D48).

Overall mortality rate was calculated by dividing the number of deaths in each capital/province in 2015 by the total population in that year, multiplied by 1000. Cause-specific mortality rates were calculated by dividing the total number of deaths from CVD or cancer in 2015 by the total population in that year, multiplied by 100,000. The total population for each capital/province in 2015 was determined using population estimates calculated by the Brazilian Institute of Geography and Statistics [29] and the National Institute of Statistics and Census of Argentina [30]. All the gender-stratified mortality rates were also calculated.

### Statistical analysis

We used average prevalence and 95% confidence intervals (95%C.I.) to describe the prevalence of behavioral risk and protective factors from 2006 to 2014 in Brazil, and from 2005 to 2013 in Argentina, stratified by the five geographical regions of each country. Analysis of Variance with repeated measures (repeated-measures ANOVA) was used to assess differences in prevalence of behavioral risk and protective factors according to the geographical regions. In addition, we assessed second-order interactions between geographical region and study year in order to check whether there was variation in temporal trends in behavioral factors between the two time-points in each country, according to region. Significant interactions characterized regional differences in temporal trends of behavioral factors.

The association of behavioral risk and protective factors with overall and cause-specific mortality rates was based on models using mean exposure across the two time-points as a summary [31, 32], and it was performed in two steps. In the first step, we used Pearson’s correlation coefficient, and correlation coefficients were classified as low (0.0–0.3), moderate (> 0.3–0.7) and high (> 0.7–1.0). Even with slowly asymmetry in outcomes distributions, we decided to use this test on the basis of the central limit theorem [33].

After that, adjusted linear regression models were tested to check whether significant associations were independent of socioeconomic and demographic factors. Linear regression models were adjusted for HDI, educational level, and gender distribution in Brazil; and for HDI, educational level, average age, and gender distribution in Argentina. We did not include average age in Brazilian models since Brazilian mortality rates were standardized according to the Argentinean population, using the direct standardization method [33]. This method allows the estimation of Brazilian mortality rates if Brazil presents the same age- and gender-structure of the Argentinean population.

In all analyses, we checked the Variance Inflation Factor (VIF) to assess collinearity in the tested models. All analyses were performed using the statistical package *Stata*, version 16.1.

## Results

### Risk factors

**Fig. 1:**
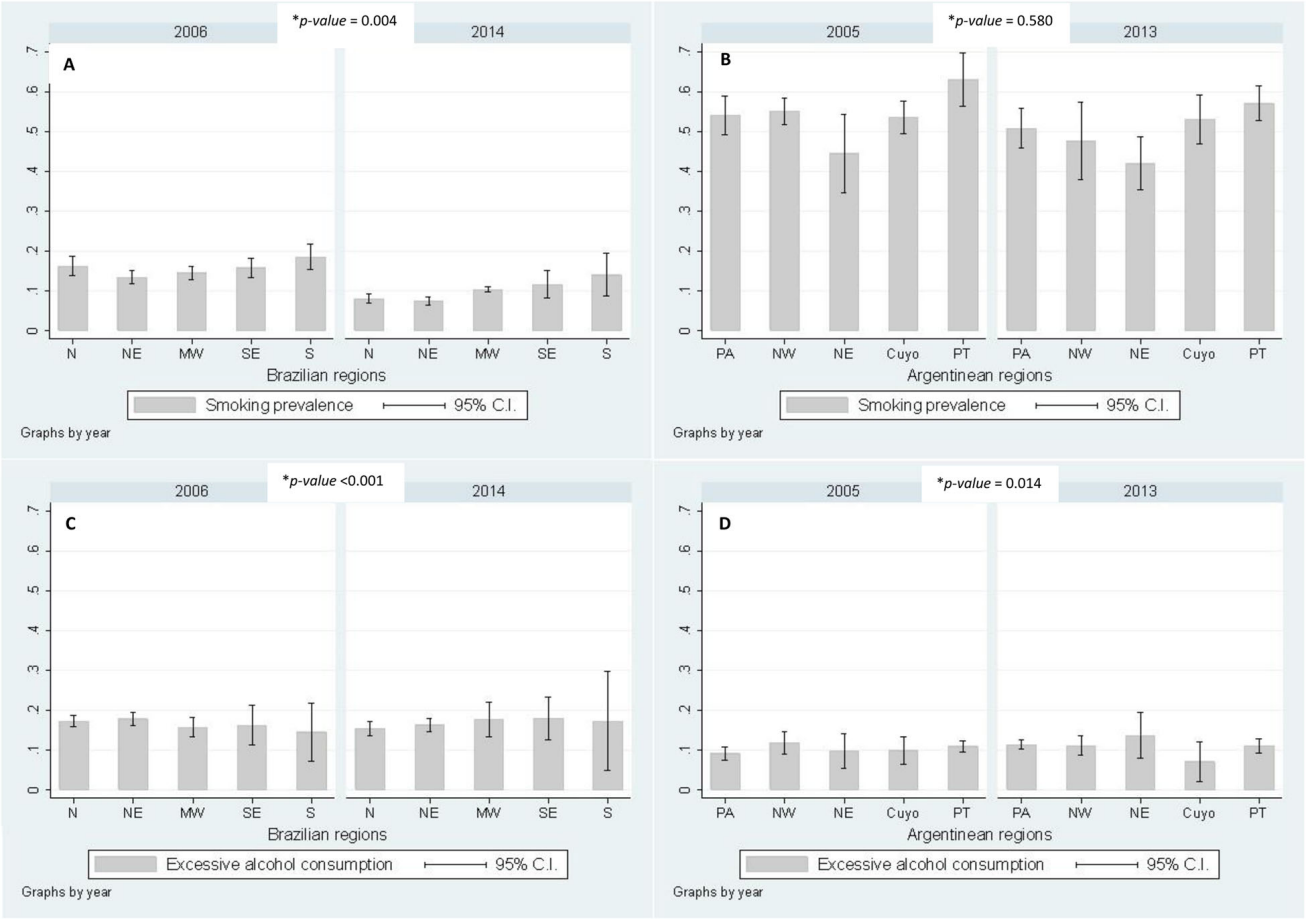
Prevalence of risk factors in Brazil and Argentina according to geographical regions (**a** - Prevalence of tobacco smoking in Brazil; **b** - Prevalence of tobacco smoking in Argentina; **c** - Prevalence of excessive alcohol consumption in Brazil; **d** - Prevalence of excessive alcohol consumption in Argentina). *p-value for second order interaction between region and survey’s year. N = North; NE = Northeast; MW = Midwest; SE = Southeast; S = South. PA = Pampeana; NW = Northwest; NE = Northeast; PT = Patagonica

There was a decrease in the prevalence of tobacco smoking in both countries. In Brazil, from 2006 to 2014, overall prevalence of tobacco smoking fell from 15.2% (95%C.I. 14.2 to 16.2%) to less than 10% (9.4%; 95%C.I. 8.3 to 10.4%). Such decrease was larger in the North region, i.e., the one which comprises Amazonian forest, while the South always presented higher prevalence (Fig. 1 a). These results were similar when stratified by gender, with the difference that prevalence of tobacco smoking was higher in men than in women; almost twice as high in 2014 (from 19.5 to 12.3% in men, and from 11.4 to 6.8% in women) (Supplementary figure1A & B).

In Argentina, prevalence of tobacco smoking has also fallen in the 8-year-period being analyzed, but that decrease was slighter: from 54.6 to 50.1% from 2005 to 2013. There was no difference in decrease in tobacco smoking according to the Argentinean regions (*p-value* for interaction = 0.580) (Fig. 1 b). As with the finding for Brazil, prevalence of tobacco smoking was higher in men than women in all regions (Supplementary figure 2A & B).

Regarding excessive alcohol consumption, this behavior was stable in both countries. In Brazil, overall prevalence was 16.8% in 2006 and 16.4% in 2014. The evolution of excessive alcohol consumption statistically differed among the Brazilian regions: the North and the Northeast had a decrease while the Midwest, the Southeast and the South, the three richest regions in the country, showed an increase (*p-value* for interaction < 0.001) (Fig. 1 c). Interestingly, even though prevalence was around three times as high in men, there was a percentage increase in this behavior in women in the 8-year-period, from 8.0% in 2006 to 8.8% in 2014, although without statistical difference (*p-value* = 0.236) (Supplementary figure 1C & D).

In Argentina, the scenario was very similar to the one in Brazil. Prevalence of overall excessive alcohol consumption was stable (it ranged from 10.4% in 2005 to 11% in 2013), with differences within Argentinean regions (Fig. 1 d). The prevalence was much higher in men (18.9% in 2005 and 18.6% in 2013) than in women (from 2.5% in 2005 to 4.1% in 2013); among women, there was an increase in excessive alcohol consumption in all regions except in *Cuyo* (Supplementary figure 2C & D).

### Protective factors

**Fig. 2:**
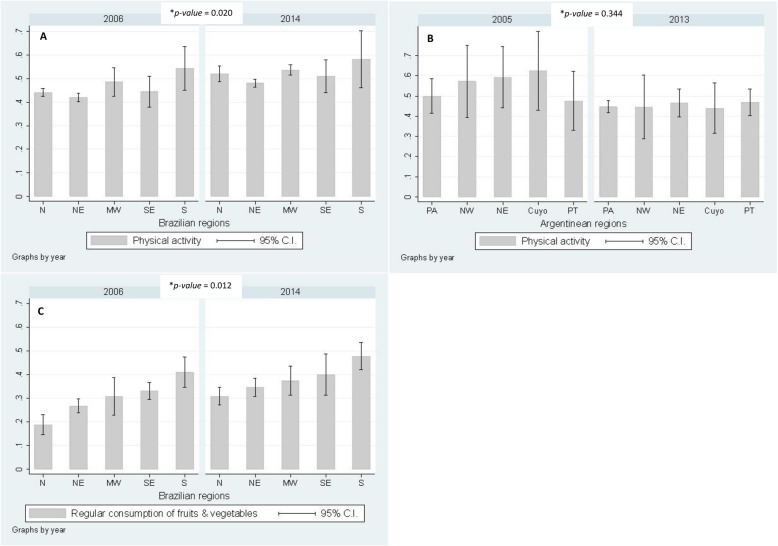
Prevalence of protective factors in Brazil and Argentina according to geographical regions (**a** - Prevalence of physical activity in Brazil; **b** - Prevalence of physical activity in Argentina; **c** - Prevalence of regular consumption of fruits and vegetables in Brazil). *p-value for second order interaction between region and survey’s year. N = North; NE = Northeast; MW = Midwest; SE = Southeast; S = South. PA = Pampeana; NW = Northwest; NE = Northeast; PT = Patagonica

There was an increase in the overall prevalence of PA from 2006 to 2014 in Brazil (from 45.3 to 51.5%). The North region had the largest increase (*p-value* for interaction = 0.020) (Fig. 2 a). When the results were stratified by gender, we found that men had higher prevalence than women, and for both males and females, such increase statistically differed among regions (Supplementary Figure 3A & B).

On the other hand, there was a decrease in prevalence of PA in Argentina from 2005 to 2013. The rate of individuals who reported practicing PA for 150 min or more per week dropped almost 10% points: from 54.5 to 45.4%. Such decrease in PA was larger in the Northeast, the Northwest and in *Cuyo* (Fig. 2 b). A similar scenario was found when the results were stratified by gender, with men presenting higher prevalence of PA but a larger decrease in this practice. In men, decreased PA was also more evident in the Northeast, the Northwest and in *Cuyo*, with no differences according to region for women (Supplementary Figure 4A & B).

Finally, regular F&V consumption increased substantially in Brazil from 2006 to 2014, from 27.8 to 36.2%. This increase statistically differed among Brazilian regions; it was sharper in the North region, where prevalence of regular consumption almost doubled in that period (*p-value* for interaction = 0.012) (Fig. 2 c). The gender-stratified results showed an increase in F&V consumption for both men and women in all Brazilian regions, but it was larger in the North region. It should be noted that regular consumption of F&V was always higher among women when compared to men (Supplementary Figure 3C & D).

### Overall and cause-specific mortality rates

Table 1 shows that overall and cause-specific mortality rates in Brazilian capitals were lower when compared to the Argentinean provinces. In addition, both countries presented a similar scenario: regional differences with richer regions presenting higher mortality rates, and CVD being the main cause of death. After standardization of Brazilian mortality rates according to the Argentinean population, Brazilian overall and cause-specific mortality rates increased. This result indicates that if Brazil had presented the same age- and gender-distribution of Argentinean population, its mortality rates would have been higher.

**Table 1:** Overall and cause-specific mortality rates in Brazilian capitals and Argentineans provinces (2015)

	Mortality rate		
	Overall (per 1000)	By cancer (per 100,000)	By CVD (per 100,000)
	Mean (95%C.I.)	Mean (95%C.I.)	Mean (95%C.I.)
**Brazil**			
**Geographical region**	*0.020*	*< 0.001*	*0.010*
North	4.7 (3.9; 5.5)	76.6 (61.9; 91.4)	85.0 (63.7; 106.2)
Northeast	6.1 (5.7; 6.4)	101.4 (91.9; 110.8)	127.3 (110.8; 143.7)
Midwest	5.5 (4.0; 7.0)	105.1 (81.4; 128.8)	119.5 (75.3; 163.7)
Southeast	6.6 (4.4; 8.7)	130.9 (110.2; 151.6)	144.1 (87.7; 200.5)
South	6.0 (2.4; 9.6)	150.0 (61.3; 238.7)	130.8 (52.5; 209.0)
**Total**	**5.7 (5.3; 6.1)**	**105.3 (94.1; 116.5)**	**118.0 (105.4; 130.7)**
**Brazil (Standardized)**^**a**^			
**Geographical region**	*0.474*	*0.176*	*0.725*
North	8.1 (6.3; 9.8)	135.1 (109.2; 161.0)	176.7 (129.7; 223.6)
Northeast	7.6 (7.1; 8.2)	127.0 (117.2; 136.9)	172.6 (146.4; 198.7)
Midwest	7.8 (6.0; 9.5)	144.4 (125.4; 163.3)	182.8 (135.7; 229.8)
Southeast	7.0 (5.1; 8.9)	135.4 (118.5; 152.3)	158.2 (98.8; 217.7)
South	6.6 (4.7; 8.5)	157.7 (107.1; 208.3)	148.2 (111.1; 185.2)
**Total**	**7.6 (4.8; 10.9)**	**136.3 (128.6; 144.1)**	**170.3 (106.8; 263.4)**
**Argentina**			
**Geographical region**	*< 0.001*	*< 0.001*	*< 0.001*
Pampeana	8.4 (7.3; 9.5)	177.8 (152.1; 203.4)	193.4 (148.6; 238.3)
Northwest	6.1 (5.6; 6.6)	93.5 (77.2; 109.9)	108.3 (73.3; 143.4)
Northeast	6.3 (5.6; 7.0)	116.3 (96.3; 136.3)	141.6 (75.6; 207.5)
Cuyo	6.9 (5.9; 7.8)	141.0 (121.9; 160.1)	176.7 (100.5; 252.9)
Patagonica	5.0 (3.7; 6.4)	117.3 (87.9; 146.8)	94.7 (67.8; 121.7)
**Total**	**6.6 (6.0; 7.2)**	**129.3 (113.9; 144.7)**	**140.8 (119.1; 162.6)**

*CVD* cardiovascular diseases, *95% C.I* 95% confidence interval

^a^Standardized according to the Argentinean population’s sex- and age-distribution

### Crude and adjusted associations between behavioral factors and mortality rates

Correlation analyses showed that only behavioral protective factors presented significant correlations with mortality rates in Brazil. There were negative and moderate correlations of F&V consumption and prevalence of PA with overall and CVD mortality. Nevertheless, stratification by gender showed that while correlation between F&V and overall and CVD mortality rates were significant for both males and females, correlation with PA was significant for women only (Table 2).

**Table 2:** Crude correlations between behavioral factors and mortality rates in Brazilian capitals and Argentinean provinces

	Brazil			Argentina		
	Overall r (*p-value*)	By cancer r (*p-value*)	By CVD r (*p-value*)	Overall (*p-value*)	By cancer r (*p-value*)	By CVD r (*p-value*)
	***Overall population***			***Overall population***		
**Risk factors**						
Tobacco smoking	−0.07 (*0.608*)	0.22 (*0.116*)	− 0.03 (*0.844*)	− 0.18 (*0.222)*	0.04 (*0.782*)	− 0.16 (*0.264*)
Excessive alcohol consumption	−0.18 (*0.199*)	− 0.08 (*0.560*)	− 0.21 (*0.134*)	−0.23 (*0.115)*	− 0.23 (*0.120*)	**−0.36 (*0.011*)**
**Protective factors**						
Consumption of F&V	**−0.49 (*< 0.001*)**	0.05 (*0.732*)	**−0.39 (*0.003*)**	–	–	–
Physical activity	**−0.28 (*0.037*)**	0.10 (*0.455*)	**−0.29 (*0.036*)**	− 0.06 (*0.757*)	−0.06 (*0.710*)	0.01 (*0.964*)
	***Males***			***Males***		
**Risk factors**						
Tobacco smoking	−0.05 (*0.725*)	0.19 (*0.167*)	0.05 (*0.736*)	−0.27 (0.059)	−0.09 (*0.545*)	**− 0.31 (*0.032*)**
Excessive alcohol consumption	−0.11 (*0.424*)	− 0.16 (*0.243*)	−0.16 (*0.239*)	− 0.24 (0.106)	**−0.29 (*0.048*)**	**− 0.43 (*0.002*)**
**Protective factors**						
Consumption of F&V	**−0.42 (*0.002*)**	0.12 (*0.380*)	**−0.32 (*0.017*)**	–	–	–
Physical activity	−0.23 (*0.089*)	0.11 (*0.418*)	−0.26 (*0.062*)	− 0.05 (0.737)	−0.12 (*0.417*)	− 0.03 (*0.821*)
	***Females***			***Females***		
**Risk factors**						
Tobacco smoking	−0.09 (*0.510*)	0.18 (*0.196*)	−0.13 (*0.343*)	−0.06 (0.704)	0.16 (*0.282*)	0.00 (*0.989*)
Excessive alcohol consumption	−0.07 (*0.633*)	0.05 (*0.706*)	−0.13 (*0.334*)	0.04 (0.783)	0.09 (*0.527*)	0.01 (*0.972*)
**Protective factors**						
Consumption of F&V	**−0.50 (*< 0.001*)**	− 0.06 (*0.674*)	**− 0.42 (*0.001*)**	–	–	–
Physical activity	**−0.28 (*0.040*)**	− 0.01 (*0.965*)	**−0.29 (*0.032*)**	− 0.02 (0.907)	0.04 (*0.765*)	0.05 (*0.761*)

*CVD* cardiovascular diseases, *F&V* fruits and vegetables, *r* Pearson’s correlation coefficient

In Argentina, however, only risk factors have been correlated with the mortality rates included in our analyses. In the overall population, there was a negative and moderate correlation between prevalence of excessive alcohol consumption and mortality from CVD. Stratification by gender showed that prevalence of excessive alcohol consumption correlated negatively and moderately with cancer and CVD mortality rates in men. In addition, prevalence of tobacco smoking also correlated negatively with CVD mortality in men (Table 2).

Adjusted linear regression models showed that prevalence of F&V consumption remained negatively associated with mortality rates in Brazilian capitals: an increase of 1% point in regular F&V consumption resulted in less 0.07 death per 1000 inhabitants. Stratification by gender showed that significant results were found for women only: an increase of 1% point in F&V consumption caused less 0.08 death per 1000 inhabitants and less 1.17 deaths by cancer per 100,000 inhabitants, regardless of capital’s HDI and education level (Table 3). In Argentina, almost all significant effects were attenuated after adjustment for confounders. Just one effect remained: surprisingly, an increase of 1% point in prevalence of excessive alcohol consumption resulted, on average, in less 6.09 CVD deaths per 100,000 inhabitants (Table 3).

**Table 3:** Adjusted linear regression models between behavioral risk and protective factors and overall and cause-specific mortality rates in Brazilian capitals and Argentinean provinces

**Brazil**			
	**Overall** β (95% C.I.)	**By Cancer** β (95% C.I.)	**By CVD** β (95% C.I.)
	***Overall population***^**a**^		
Consumption of F&V	**−0.07 (− 0.13; − 0.02)**	−0.88 (−1.85; 0.10)	−1.64 (−3.38; 0.10)
	–		
Physical activity	0.00 (−0.09; 0.10)	0.28 (− 1.30; 1.85)	− 0.08 (−2.90; 2.75)
***Male***^**b**^			
Consumption of F&V	−0.03 (− 0.09; 0.02)	−0.36 (− 1.43; 0.70)	−0.66 (− 2.47; 1.15)
Physical activity	− 0.02 (− 0.09; 0.06)	0.48 (− 1.00; 1.95)	−0.84 (− 3.35; 1.66)
***Female***^**b**^			
Consumption of F&V	**−0.08 (− 0.14; − 0.03)**	**−1.17 (− 2.09; − 0.25)**	−1.62 (− 3.26; 0.02)
Physical activity	−0.07 (− 0.15; 0.01)	−0.76 (− 2.15; 0.62)	1.20 (−3.61; 1.21)
**Argentina**			
	**Overall** β (95% C.I.)	**By Cancer** β (95% C.I.)	**By NCD** β (95% C.I.)
***Overall population***^**c**^			
Tobacco smoking	−0.01 (−0.04; 0.02)	− 0.19 (− 0.99; 0.62)	−1.38 (−3.07; 0.32)
Excessive alcohol consumption	−0.01 (− 0.09; 0.07)	1.27 (− 0.77; 3.32)	− 3.57 (−7.94; 0.80)
***Males***^d^			
Tobacco smoking	− 0.01 (− 0.04; 0.02)	−0.39 (− 1.29; 0.51)	− 2.96 (−5.97; 0.03)
Excessive alcohol consumption	− 0.01 (− 0.06; 0.05)	0.62 (− 1.03; 2.26)	**−6.09 (− 11.48; − 0.70)**
***Females***^d^			
Tobacco smoking	−0.04 (− 0.08; 0.00)	−0.33 (− 1.08; 0.42)	−2.55 (− 5.80; 0.69)
Excessive alcohol consumption	− 0.08 (− 0.25; 0.10)	1.11 (− 2.32; 4.55)	−4.77 (− 19.96; 10.42)

*CVD* cardiovascular diseases, *F&V* fruits and vegetables, *95% C.I* 95% confidence interval

^a^Adjusted for Human Development Index, education and gender distribution

^b^Adjusted for Human Development Index and education

^c^Adjusted for Human Development Index, education, age and gender distribution

^d^Adjusted for Human Development Index and education and age

## Discussion

This ecological study, which assessed temporal trends in behavioral risk and protective factors and their association with mortality rates in two of the biggest countries in South America, has revealed interesting and important results from a public health perspective. The most relevant finding was a negative association between regular F&V consumption and mortality rates in Brazilian capitals. The Brazilian cities with higher prevalence of regular F&V consumption between 2006 and 2014 presented lower overall mortality rates, regardless of HDI and educational level. Our study also revealed that this association was only found among women after gender stratification. Results for women also showed that higher prevalence of regular F&V consumption was negatively associated with mortality from cancer.

Previous studies have indicated positive effects of F&V consumption on mortality rates [34–36]. A recent meta-analysis of 16 prospective cohort studies showed an inverse association between higher intake of F&V and overall and CVD mortality [36]. Additionally, a study conducted in Australia showed a negative association between F&V consumption and all-cause mortality [35], while a study from Eastern Europe showed that F&V intake was inversely associated with stroke mortality [37].

Data from 18 countries, including Brazil and Argentina, also showed an inverse association between intake of fruits, vegetables and legumes, and total and cardiovascular mortality. Nevertheless, no association was found when only South American countries were analyzed [38]. In Brazil, CVD and all-cause mortality attributable to dietary intake increased from 1980s to 2009, but it was related to increases in consumption of ultraprocessed food and decreases in F&V consumption in this period [39].

Importantly, unlike the above-mentioned research studies, our study detected such association at the populational level. The negative association between F&V consumption and mortality rates at the ecological level can be partly explained by the increase in the prevalence of F&V consumption in Brazilian capitals in the last decade. From 2006 to 2014, prevalence of regular F&V consumption in Brazil increased more than 30%, and consumption was always higher among women. This increase may be result of several strategies adopted in Brazil to improve dietary intake patterns of the Brazilian population, e.g., the National Food and Nutrition Policy launched over 20 years ago [40], the Food Purchase Program, the National School Feeding Program [41], in addition to the Global Strategy for Healthy Eating, Physical Activity and Health [42] and the National Health Promotion Policy [43]. Moreover, Brazil was one of the first countries to implement a food guide for its population in 2006, and in 2014, the Brazilian Ministry of Health launched an updated food guide focusing on a reduction in the consumption of ultraprocessed foods [44]. Therefore, the positive impact of F&V consumption on mortality rates seen in our study may be a consequence of these above-mentioned strategies adopted in the last years, which increased regular F&V consumption by the Brazilian population.

Brazil also succeeded in increasing prevalence of PA in the 8-year-period covered by our study. In 2014, more than 50% of adults living in Brazilian capitals reported at least 150 min of PA per week. In contrast, there was a reduction in PA in Argentina from 2005 to 2013. Differences in frequency of PA can be explained by differences in strategies to increase this practice in the two countries. Implementation of programs to encourage PA as well as mobilization and support for this practice have been some of the priority strategies for health promotion in Brazil since the early 2000s. Along with the implementation of the Global Strategy for Healthy Eating, Physical Activity and Health in 2004 [42] and the National Health Promotion Policy in 2006 [43], two other initiatives were undertaken: the Family Health Support Center was created to support the consolidation of primary health care in Brazil [45], and the Health Academies Program was launched to create public places with infrastructure, equipment and qualified professionals in order to promote self-care and support PA [46]. All these strategies and programs have helped Brazil to increase its prevalence of PA, mainly in the state capitals.

In Argentina, on the other hand, policies to include and support PA started to be implemented a little later, more specifically at the beginning 2010s, when the Healthy Argentina National Plan (*Plan Nacional Argentina Saludable*) was created. This policy was reinforced by the creation of the National Program to Fight Against Sedentarism in 2013 as well as the National Plan for Healthy Eating in childhood and adolescence for prevention of overweight and obesity (ASI Plan) in 2019 [47]. Perhaps the effects of these strategies were not captured by our study, as we have observed a decrease in PA from 2005 to 2013 in Argentina, when the policies were just starting to be implemented. Nevertheless, data from the 2018 ENFR showed an increase in PA in Argentina [48], which may be indicative of a positive effect of these policies on prevalence of PA.

The positive impact of different types of PA on mortality rates has been found for a cohort study with 130,000 individuals from high-income, middle-income and low-income countries. In middle-income countries, e.g., Brazil and Argentina, moderate and high PA were associated with lower risk of mortality and major CVD [49]. In our study, the increased prevalence of PA observed from 2006 to 2014 resulted in lower overall and cancer mortality rates in Brazilian women. Nevertheless, the effect has been attenuated after adjustment for HDI and educational level. This attenuation might have been due to socioeconomic differences among Brazilian capitals: those capitals with higher PA rates are precisely those with higher HDI and educational level, where mortality rates by NCD are also higher.

Regarding tobacco smoking, prevalence reduced by almost 40% from 2006 to 2014 in Brazil (from 15.2 to 9.4%). In comparison, the reduction of tobacco smoking in Argentina was much slighter: a decrease of only 8% (from 54.6 to 50.1%). However, how has Brazil achieved such a positive result in prevalence of tobacco smoking? Since 2003, when Brazil adopted the Tobacco free initiative MPOWER, the country has taken several actions to reduce this behavior; for example, ban on smoking in public places and means of transportation, smoking cessation treatment, warnings on cigarette packages, ban on cigarette advertisements in the media and at points of sale, and cigarette tax that accounts for more than 80% of the total price of cigarettes, which establishes Brazil as the country with the highest cigarette tax of all members in the Americas region [13].

On the other hand, tobacco smoking rates in Argentina are still some of the highest in South America [50], despite the reduction from 2005 to 2013 observed in our study. Tobacco free initiatives adopted in Argentina were much more modest when compared to Brazil, which may explain differences in tobacco reduction in both countries. In Argentina, the WHO Framework Convention on Tobacco Control was also signed in 2003 but it has not been ratified again. Only in 2011 did the country approve Law number 26,687, which regulates advertising, promotion and consumption of tobacco products and provides a free smoke environment. The cost of cigarettes in Argentina was among the cheapest in the world (around 1 USD a pack of 20 cigarettes) until 2016, when the tax on cigarettes and other tobacco by-products was increased [51]. Even so, the Argentinean legislation has important gaps; for example, it allows advertising at points of sale and commercial publications for people or institutions that participate in the tobacco production and consumption chain [48].

Both countries presented a similar scenario in trends of excessive alcohol consumption in the last decade: heterogeneity among regions, higher prevalence among men but a sharper increase among women. Although, historically, women have consumed less alcohol than men, this scenario is quickly changing around the world. In Europe, for example, alcohol consumption is increasing gradually among young women [52]. This change in women’s behavior can be attributed to the evolution of women’s role in the society of the twenty-first century, with greater gender equality.

While previous studies have demonstrated a link between excessive alcohol consumption and mortality [53–55], we were not able to find any association in Brazil. In Argentina, however, an intriguing association remained significant after adjustment for confounders: those Argentinean provinces with higher prevalence of excessive alcohol consumption presented lower mortality from CVD. Moderate wine consumption has been suggested to improve cardiovascular health [56, 57] and wine has been declared a national drink in Argentina (Law 26.870/13) and exempt of taxes imposed to other alcoholic beverages, which can stimulate its consumption. As the questionnaire used in ENFR measured self-reported alcohol consumption, people might have overestimated their consumption, and this could be a reason for this intriguing result seen in our study.

Unavailability to assess regular F&V consumption in Argentina was the biggest limitation of our study. The questions regarding F&V consumption in the 2005 and 2013 ENFR were different; for this reason, they do not allow comparisons between the two time-points evaluated here. In addition, self-reported information for all behavioral factors analyzed may be considered another limitation of our study. Nevertheless, self-reported information about important risk factors (such as tobacco smoking, alcohol consumption and dietary intake) presents good reliability in epidemiological studies [58].

Ecological studies use group-based data, which results in loss of information or concealment of details at the individual level. An additional problem related to ecological studies is the heterogeneity of exposures or covariates within groups, which is not fully captured due to the group-based distribution [59]. Therefore, results showed in our study must be carefully interpreted as the observed associations may differ from the associations at individual level within groups of the same population. Nevertheless, we do not believe in ecological fallacy here since the positive effects of F&V consumption on mortality have already been demonstrated in individual-level investigations, as we described above. A last limitation of our study is the low proportion of Brazilian households with landline telephones (35% in 2014), since Vigitel is a survey conducted by phone. However, Vigitel deals with this issue using sampling weights to adjust the sample distribution with landline telephone to the reference population [60, 61]. Finally, despite all the limitations, analyses based on national population surveys from two of the biggest Latin American countries, studying an association hardly explored in both countries, can be considered a strength of our study.

## Conclusion

In conclusion, prevalence of regular F&V consumption increased in Brazilian capitals and was associated with lower overall mortality rate. This result suggests a positive impact of Brazilian policies to improve dietary intake patterns on mortality, mainly in women, for whom F&V consumption was also associated with cancer mortality. Additionally, our study also showed improvement in prevalence of PA and tobacco smoking, particularly in Brazil. Approaches focusing on reduction of tobacco smoking and sedentarism are especially needed in Argentina in order to reach similar results of those seen in Brazil.

## Supplementary information

**Additional file 1: Supplementary figure 1.** Prevalence of risk factors in Brazil according to geographical regions and stratified by gender.

**Additional file 2: Supplementary figure 2.** Prevalence of risk factors in Argentina according to geographical regions and stratified by gender.

**Additional file 3: Supplementary figure 3.** Prevalence of protective factors in Brazil according to geographical regions and stratified by gender.

**Additional file 4: Supplementary figure 4.** Prevalence of physical activity in Argentina according to geographical regions and stratified by gender.

## Data Availability

All data from VIGITEL and ENFR are publicly available. Data from VIGITEL can be obtained at http://svs.aids.gov.br/bases_vigitel_viva/vigitel.php, while ENFR data can be assessed at http://www.indec.gov.ar/bases-de-datoss.asp. Mortality information from both countries are also publicly available. Brazilian mortality information can be assessed at http://tabnet.datasus.gov.br/cgi/deftohtm.exe?sim/cnv/obt10uf.def. Argentinean mortality information can be gathered in the Department of Statistics and Health Information of the Ministry of Health (http://www.deis.msal.gov.ar/).

## Abbreviations

CVD: Cardiovascular diseases
95%C.I.: 95% Confidence intervals
ENFR: National Survey of Risk Factors
F&V: Fruits and vegetables
GDP: Gross domestic product
HDI: Human Development Index
ICD-10: International Classification of Diseases – 10th revision
LMIC: Low- and middle-income countries
NCD: Non-communicable diseases
Repeated-measures ANOVA: Analysis of variance with repeated measures
VIF: Variance inflation factor
WHO: World Health Organization
