# Supplementary material for: Temporal trends in behavioral risk and protective factors and their association with mortality rates: results from Brazil and Argentina
Source: BMC Public Health. 2020 Sep 11;20:1390. doi: 10.1186/s12889-020-09512-9 (PMC7488766; doi:10.1186/s12889-020-09512-9)
Supplement: Supplementary file 2 — Additional file 2: Supplementary figure 2. Prevalence of risk factors in Argentina according to geographical regions and stratified by gender. [file 12889_2020_9512_MOESM2_ESM.docx]

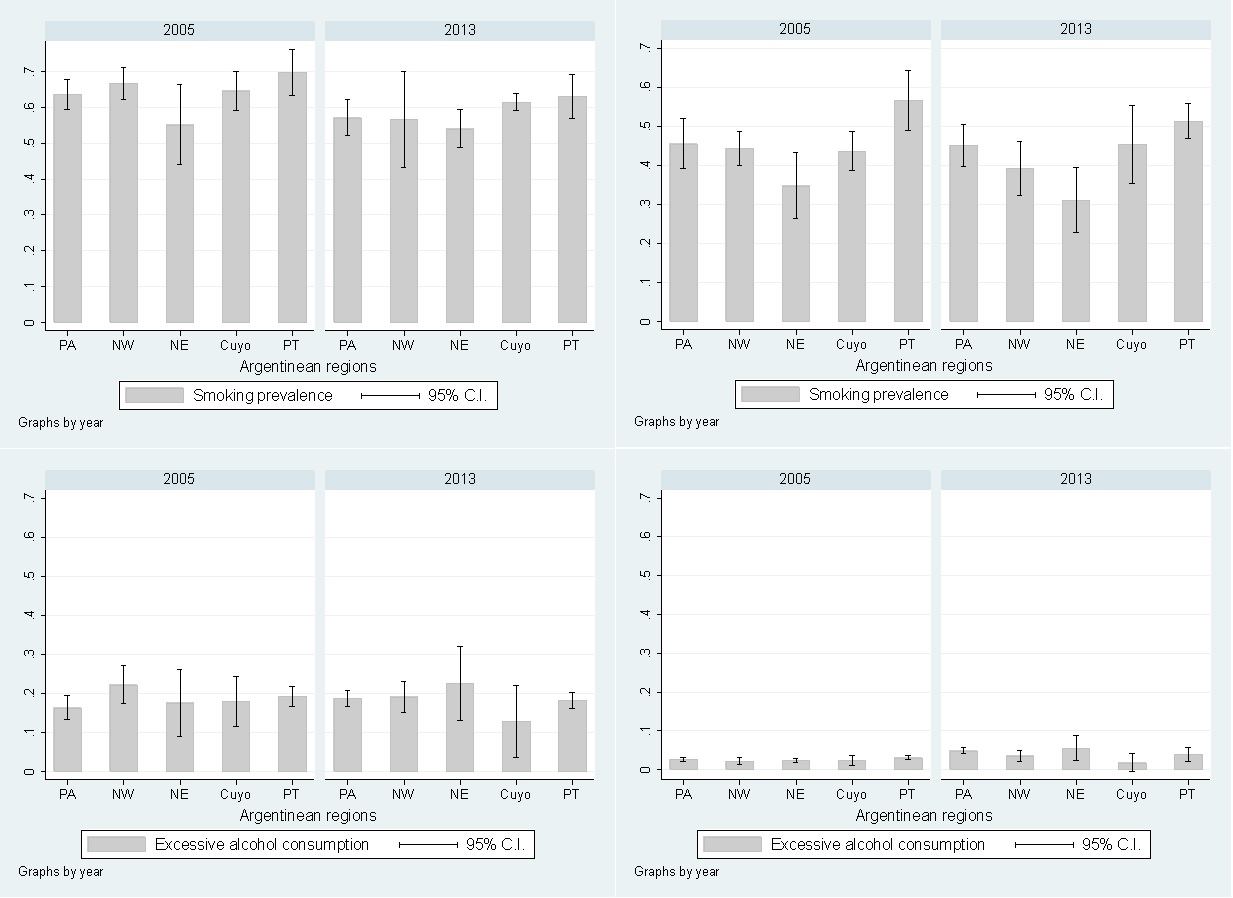


**p-value* = 0.026

**p-value* = 0.028

**D**

**C**

**B**

**p-value* = 0.410

**p-value* = 0.520

**A**

**Supplementary figure 2.** Prevalence of risk factors in Argentina according to geographical regions and stratified by gender (A - Prevalence of tobacco smoking in men; B - Prevalence of tobacco smoking in women; C - Prevalence of excessive alcohol consumption in men; D - Prevalence of excessive alcohol consumption in women).

**p-value* for second order interaction between region and survey’s year.

PA = Pampeana; NW = Northwest; NE = Northeast; PT = Patagonica
