# Supplementary material for: Temporal trends in behavioral risk and protective factors and their association with mortality rates: results from Brazil and Argentina
Source: BMC Public Health. 2020 Sep 11;20:1390. doi: 10.1186/s12889-020-09512-9 (PMC7488766; doi:10.1186/s12889-020-09512-9)
Supplement: Supplementary file 3 — Additional file 3: Supplementary figure 3. Prevalence of protective factors in Brazil according to geographical regions and stratified by gender. [file 12889_2020_9512_MOESM3_ESM.docx]

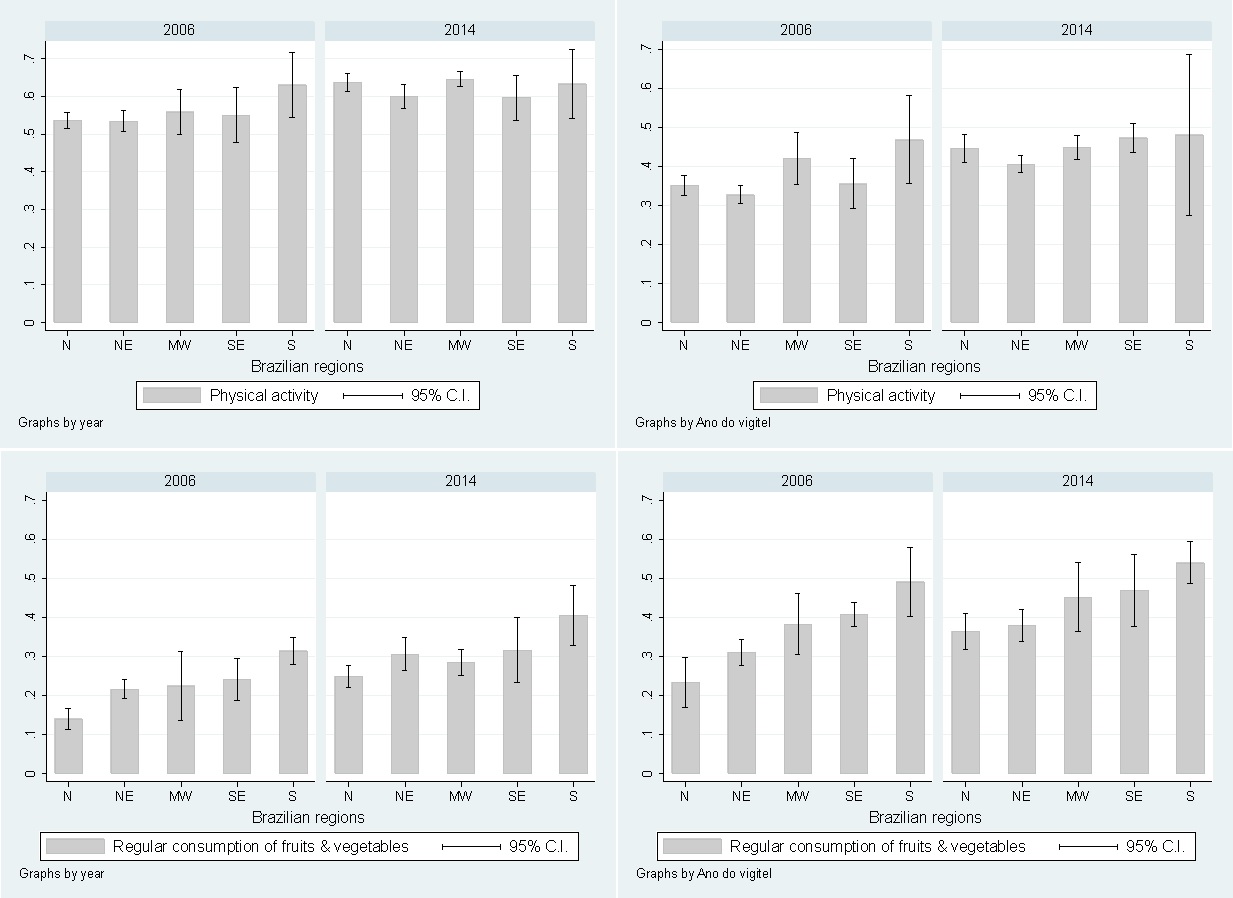


**p-value* = 0.007

**p-value* = 0.227

**D**

**C**

**p-value* = 0.013

**p-value* = 0.012

**B**

**A**

**Supplementary figure 3.** Prevalence of protective factors in Brazil according to geographical regions and stratified by gender (A - Prevalence of physical activity in men; B - Prevalence of physical activity in women; C - Prevalence of regular consumption of fruits and vegetables in men; D - Prevalence of regular consumption of fruits and vegetables in women).

**p-value* for second order interaction between region and survey’s year.

N = North; NE = Northeast; MW = Midwest; SE = Southeast; S = South
